# Effectiveness of mindfulness-based stress reduction for depression in post-stroke patients: a systematic review and meta-analysis

**DOI:** 10.3389/fpsyt.2026.1809626

**Published:** 2026-05-08

**Authors:** Ying Sun, Shirong Bo, Yongsheng Li

**Affiliations:** 1 Qingdao Municipal Hospital, Qingdao, China; 2 The Affiliated Hospital of Qingdao University, Qingdao, China

**Keywords:** depressive symptoms, meta-analysis, mindfulness-based stress reduction, stroke, systematic review

## Abstract

**Background:**

Depression is a common complication after stroke. Although prior studies suggest that mindfulness-based stress reduction (MBSR) may alleviate depressive symptoms in post-stroke patients, the evidence remains inconclusive. This meta-analysis aimed to synthesize randomized controlled trials (RCTs) to evaluate the effects of MBSR on depressive symptoms after stroke.

**Methods:**

PubMed, Web of Science, Embase, the Cochrane Library, PsycINFO, CNKI, and Wanfang Data were searched from inception to October 2025. Eligible studies were RCTs assessing the effects of MBSR on depressive symptoms in post-stroke patients. Two reviewers independently performed study selection, data extraction, and risk-of-bias assessment.

**Results:**

Eight RCTs involving 469 participants were included. Meta-analysis using the standardized mean difference (SMD) showed that MBSR significantly reduced depressive symptoms compared with control conditions (SMD = −0.96, 95% CI: −1.35 to −0.58, P < 0.001), with substantial heterogeneity (I² = 71%). The 95% prediction interval ranged from −1.95 to 0.03, indicating that the true effect may be negligible in some future settings. In subgroup analyses, trials conducted in Asia demonstrated significant benefits (SMD = −1.27, 95% CI: −1.60 to −0.93, P < 0.001), whereas trials conducted in other regions did not reach statistical significance (SMD = −0.31, 95% CI: −0.74 to 0.13, P = 0.17); the difference between subgroups was significant (Q-between = 11.77, P = 0.0006). Participants receiving MBSR during the stable post-stroke phase showed significant improvement (SMD = −1.02, 95% CI: −1.57 to −0.47, P < 0.001), whereas those treated during the acute phase did not (SMD = −0.80, 95% CI: −1.88 to 0.27, P = 0.14); the subgroup difference was not significant (Q-between = 0.13, P = 0.7204).

**Conclusions:**

MBSR may reduce depressive symptoms after stroke, particularly during the stable phase. However, the overall certainty of evidence was rated as low due to high risk of bias, substantial heterogeneity, and imprecision. Well-designed RCTs with longer follow-up are warranted to confirm these results.

**Systematic Review Registration:**

https://www.crd.york.ac.uk/prospero/, identifier CRD420251271797.

## 1 Introduction

Stroke is a major global public health challenge (1). Among non-communicable diseases, it ranks as the second leading cause of death and the third leading cause of disability worldwide, with its annual global economic burden estimated to exceed USD 890 billion (2). Owing to its long-term impact on functional outcomes, quality of life, and healthcare systems, stroke-related complications have become an important clinical issue (3).

Depression is among the most common complications after stroke (4). Previous studies indicate that approximately 53% of stroke patients experience depressive symptoms at one or more time points following stroke onset (5, 6). In a large cohort of 25,488 patients, 31% developed depressive symptoms within five years after stroke, and 39%–52% reported recurrent depressive episodes (7). An observational study showed that post-stroke depression was significantly associated with lower quality of life and greater physical disability, and that persistent depression was associated with increased mortality (8). A 2024 study also found that depression increased the risk of suicide in patients with stroke (9). Collectively, these findings suggest that post-stroke depression is not only highly prevalent but also closely associated with poorer rehabilitation outcomes and adverse prognosis (10).

Current treatment strategies for post-stroke depression (PSD) mainly include pharmacotherapy and psychotherapy (11). However, existing evidence suggests that pharmacotherapy yields only modest-to-moderate symptom improvement (12, 13) and may be accompanied by clinically relevant adverse effects (14). A 2023 meta-analysis including 65 randomized controlled trials and 5,831 participants showed that, compared with placebo, pharmacotherapy was associated with a higher incidence of central nervous system and gastrointestinal adverse effects (15). Moreover, long-term use may affect neurological function and impede post-stroke recovery (16). Therefore, identifying effective and safe non-pharmacological alternatives remains a major research priority. Among psychotherapeutic approaches, mindfulness-based stress reduction (MBSR) has attracted increasing attention for PSD owing to its feasibility and favorable safety profile.

MBSR is a structured mindfulness-based intervention (17). It follows a standardized curriculum and is typically delivered in a group format over 8–10 weeks, aiming to reduce stress and alleviate psychological distress such as depression and anxiety (18). Compared with other therapeutic approaches, MBSR places greater emphasis on awareness of present-moment experience and encourages individuals to observe their thoughts, emotions, and bodily sensations with an attitude of acceptance and nonjudgment (19). This process may help reduce over-engagement with stress-related cues, maladaptive behavioral patterns, and cognitive biases, while promoting acceptance of illness-related changes (20). Consequently, MBSR may help improve negative emotional experiences and quality of life (21). Systematic reviews further suggest that mindfulness-based interventions are feasible among stroke survivors, which also provides a rationale for further evaluating the application of MBSR in post-stroke depression (22).

Existing studies suggest that MBSR may help alleviate depressive symptoms in patients with PSD (23–25). However, the current evidence remains insufficient, and the findings are not fully consistent. A clinical study in patients with chronic stroke did not observe a significant improvement in depressive symptoms following MBSR (26). Meanwhile, a previous meta-analysis combined MBSR with mindfulness-based cognitive therapy, thereby precluding an independent evaluation of the effect of MBSR alone on PSD (27). In addition, prior studies have shown substantial heterogeneity in intervention protocols, follow-up duration, and study quality (28). Therefore, the present study advances prior research in three key aspects: (1) it focuses exclusively on MBSR as a standalone intervention, rather than combining it with other mindfulness-based approaches; (2) it incorporates the most recent evidence from trials published through 2025; and (3) it systematically examines potential moderators—including geographic region, recruitment setting, and post-stroke stage—that may explain heterogeneous findings across previous studies.

## 2 Methods

### 2.1 Protocol and registration

This systematic review and meta-analysis was conducted in accordance with the Preferred Reporting Items for Systematic Reviews and Meta-Analyses (PRISMA) Statement. The study protocol was registered in the PROSPERO database (registration number: CRD420251271797).

### 2.2 Search strategy

We systematically searched PubMed, Web of Science, Embase, the Cochrane Library, PsycINFO, CNKI, and Wanfang from inception to October 2025. Both controlled vocabulary (e.g., MeSH/Emtree terms) and free-text keywords were used. For each concept, synonymous terms were combined using the Boolean operator “OR”. Stroke-related terms included stroke, apoplexy, apoplectic, post-stroke, cerebral infarction, cerebral hemorrhage, intracerebral hemorrhage, brain ischemia, brain infarction, cerebrovascular disorder, cerebrovascular accident, and cerebrovascular disease. MBSR-related terms included meditation, mindfulness, mindfulness-based stress reduction, and MBSR. To maximize retrieval, we also manually screened the reference lists of included studies and relevant reviews to identify additional eligible studies. The search strategy was intentionally broad to maximize the identification of MBSR-related studies in stroke populations, after which studies specifically focusing on post-stroke depression were identified during the study selection process.

### 2.3 Inclusion and exclusion criteria

Eligibility criteria were defined according to the PICOS framework (Population, Intervention, Comparison, Outcomes, and Study design).

Inclusion criteria: (1) study design: randomized controlled trials; (2) participants: patients diagnosed with stroke according to recognized diagnostic criteria; (3) intervention: mindfulness-based stress reduction (MBSR) or modified MBSR; (4) comparison: non-MBSR conditions, such as usual care, no treatment, health education, sham intervention, or other control conditions without an MBSR component; and (5) outcomes: studies reported depression-related outcome measures after the intervention and provided sufficient data for effect size calculation.

Exclusion criteria: (1) non-randomized studies; (2) studies that did not report depression-related outcome measures; (3) studies in which the independent effect of MBSR could not be evaluated; (4) conference abstracts, dissertations or theses, book chapters, reviews, duplicate publications, or animal studies; and (5) studies with insufficient data for analysis even after attempts to contact the authors.

### 2.4 Data extraction

Two reviewers (Y.S. and S.R.B.) independently extracted data from each eligible study using a standardized extraction form developed for this review, in accordance with guidance from the Cochrane Handbook. Discrepancies were resolved through discussion and, when necessary, consultation with a third reviewer (Y.S.L.). Extracted information included the first author, publication year, country, participant characteristics, sample size, baseline demographic and clinical characteristics, post-stroke stage, details of the intervention and comparator conditions, assessment time points, and outcome measures. Quantitative data were extracted to calculate effect sizes. When relevant data were missing or incompletely reported, the corresponding authors were contacted by email to request additional information. The detailed study selection process, intervention characteristics, and extracted data are provided in the **Supplementary Materials** (**Supplementary Tables S1**–**S3**).

### 2.5 Risk of bias

Two reviewers (Y.S. and S.R.B.) independently assessed the risk of bias of the included randomized controlled trials using the Cochrane Risk of Bias tool (RoB 1.0) (29). Inter-reviewer agreement was assessed using Cohen’s kappa, which indicated substantial agreement (κ = 0.85). The following domains were evaluated: random sequence generation, allocation concealment, blinding of participants and personnel (performance bias), blinding of outcome assessors (detection bias), incomplete outcome data (attrition bias), selective reporting (reporting bias), and other potential sources of bias. Each domain was judged as having a low, unclear, or high risk of bias. Discrepancies were resolved through discussion and, when necessary, adjudicated by a third reviewer (Y.S.L.).

### 2.6 Data synthesis and statistical analysis

Statistical analyses were performed using Stata (Version 17.0). Because depressive symptoms were assessed using different instruments across studies, pooled effect sizes were calculated as standardized mean differences (SMDs) with 95% confidence intervals (CIs). Given the expected clinical and methodological heterogeneity across studies, a random-effects model was prespecified for meta-analysis. Statistical heterogeneity was assessed using Cochran’s Q test and quantified using the I² statistic. In accordance with conventional thresholds, I² values of 25%–50%, 50%–75%, and >75% were interpreted as low, moderate, and high heterogeneity, respectively (30). When multiple post-baseline assessment time points were reported, only post-intervention data were included in the primary meta-analysis. For analyses conducted under the random-effects model, 95% prediction intervals were additionally calculated where appropriate. Subgroup analyses were conducted to explore potential sources of heterogeneity, and between-subgroup differences were assessed using the Q test for subgroup differences. Sensitivity analyses were performed to assess the robustness of the pooled estimates. Because fewer than 10 studies were included, publication bias was not assessed using funnel plots (31).

## 3 Results

### 3.1 Study selection

**Figure 1:**
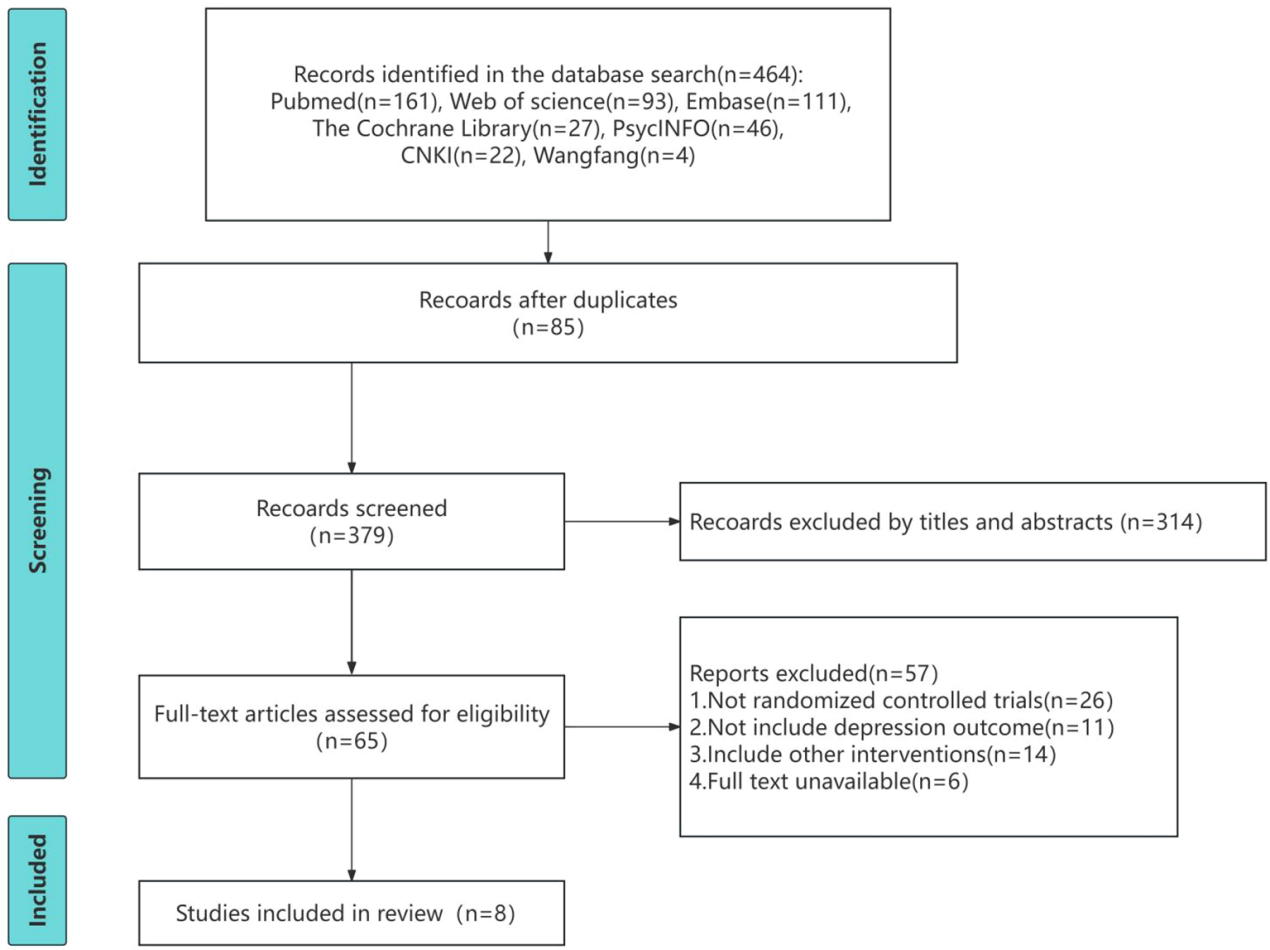
PRISMA (preferred reporting items for systematic reviews and meta-analyses) flow diagram of the study selection process.

The study selection process is illustrated in the PRISMA flow diagram (**Figure 1**). A total of 464 records were initially identified through database searching. After 85 duplicate records were removed using EndNote X9, 379 records remained. Following title and abstract screening, 314 records were excluded. The remaining 65 studies underwent full-text review, of which 57 were excluded for the following reasons: not randomized controlled trial design (n = 26), absence of depression-related outcome measures (n = 11), inclusion of additional interventions that precluded independent evaluation of the effect of MBSR (n = 14), and full-text unavailability (n = 6). Ultimately, eight studies (32–39) were included in the meta-analysis.

### 3.2 Characteristics of included trials

The characteristics of the studies included in the meta-analysis are presented in **Table 1**. A total of eight studies published between 2012 and 2025 were included (32–39), comprising 469 participants. Two studies were conducted in the United States (32, 35), one in Sweden (34), and the remaining studies in China (33, 36–39). All eight studies applied MBSR as the intervention. In five studies, the control group received usual care (35–39); one study used brain health education as the control condition (32), one used sham rTMS stimulation (33), and one did not receive any additional intervention (34). With the exception of Wu (39), which implemented a 6-week MBSR program, and Zhang and Pang (37, 38), which did not report the intervention duration, the other five studies delivered an 8-week psychological intervention (32–36). All studies enrolled participants who met recognized diagnostic criteria for stroke. Five studies included patients in the stable phase after stroke (32–34, 37, 38), two included patients in the acute phase (35, 36), and one did not report stroke phase (39). All studies used standardized assessment scales to evaluate changes in depressive symptoms before and after the intervention. In addition, two studies reported cognition-related outcomes (33, 35).

**Table 1:** Study characteristics.

Author (year)	Country, recruitment setting	Participants’ demographics	Intervention group	Control group	Duration of mindfulness intervention	Post-stroke stage	Assessment time points	Scale
Johansson et al. (34)	Sweden, Outpatient clinic	26 adults (15 males,11 females), mean age (55.4 ± 6.69) yrs	MBSR	No treatment	8 weeks	stable stage	Baseline, Post-intervention	CPRS
Baldo et al. (32)	USA, Outpatient clinic	25 adults(15 males,10 females), mean age (66.05 ± 10.75) yrs	MBSR	BHE	8 weeks	stable stage	Baseline, Post-intervention	GDS
Sophia et al. (35)	USA, Outpatient clinic	30 adults (15 males,15 females), mean age (64.7 ± 13.6) yrs	MBSR	Usual care	8 weeks	acute stage	Baseline, Post-intervention	PHQ; MoCA
Duan et al. (33)	China, Inpatient department	48 adults (39 males,9 females), mean age (55.53 ± 13.64) yrs	MBSR	sham rTMS stimulation	8 weeks	stable stage	Baseline, Post-intervention	HAMD; PSQI; MMSE
Huang et al. (36)	China, Inpatient department	80 adults (64 males,16 females) mean age (59.83 ± 6.79) yrs	MBSR	Usual care	8 weeks	acute stage	Baseline, Post-intervention	SDS; SAS
Zhang et al. (37)	China, Inpatient department	96 adults (56 males,40 females), mean age (58.99 ± 7.41) yrs	MBSR	Usual care	Not reported	stable stage	Baseline, Post-intervention	SDS; SAS
Pang et al. (38)	China, Inpatient department	80 adults (49 males,31 females), mean age (53.73 ± 11.06) yrs	MBSR	Usual care	Not reported	stable stage	Baseline, Post-intervention	SDS; HAMD;
Wu et al. (39)	China, Inpatient department	82 adults (50 males,32 females), mean age (59.75 ± 5.6) yrs	MBSR	Usual care	6 weeks	Not report	Baseline, Post-intervention	SCL-90

MBSR, Mindfulness-Based Stress Reduction; CPRS, Comprehensive Psychopathological Rating Scale; BHE, Brain Health Education; PHQ, Patient Health Questionnaire; MoCA, The Montreal Cognitive Assessment; rTMS, repetitive Transcranial Magnetic Stimulation; HAMD, Hamilton Depression Scale; PSQI, the Pittsburgh Sleep Quality Index; MMSE, a Mini-Mental State Examination; SDS, Self-Rating Depression Scale; SAS, Self-Rating Anxiety Scale; SCL-90, Symptom Checklist-90.

### 3.3 Risk of bias and quality evaluation

**Figure 2:**
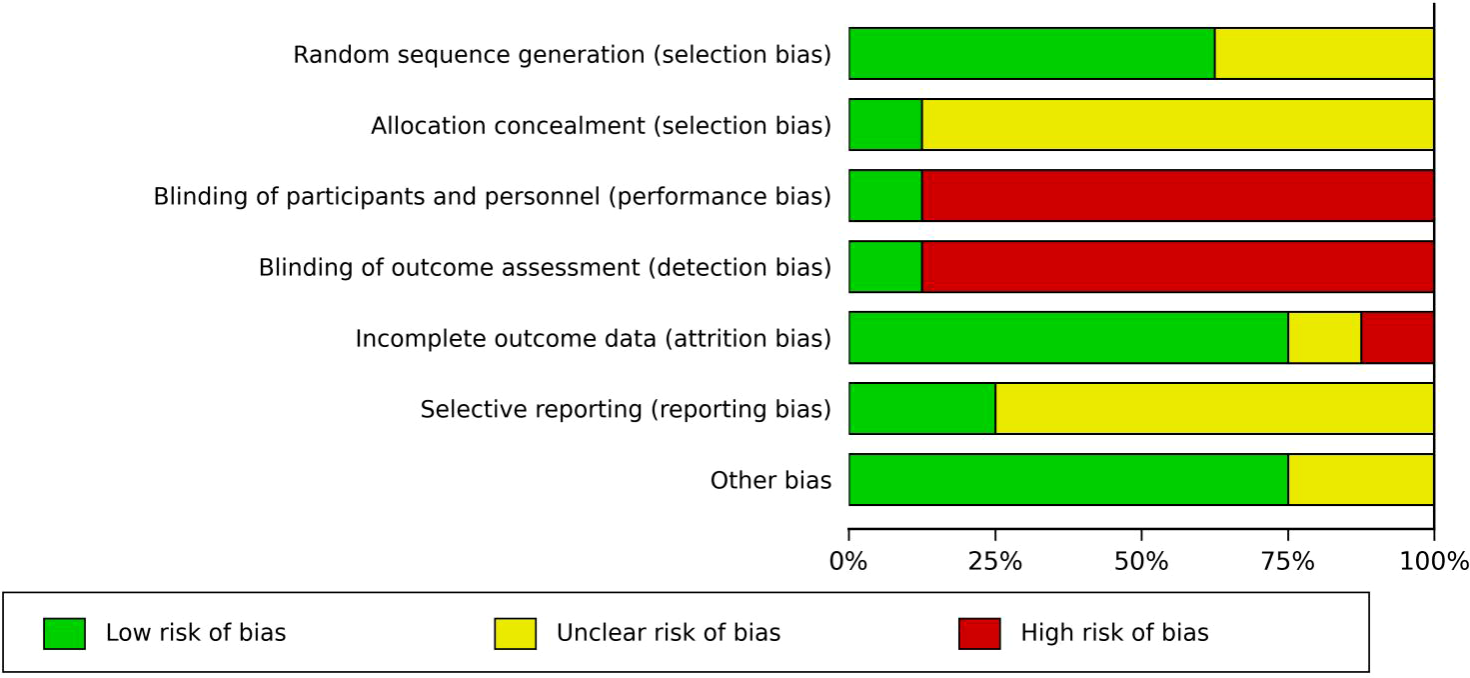
Cochrane risk of bias graph. The risk of bias graph presents ratings for all 8 studies included in the systematic review.

**Figure 3:**
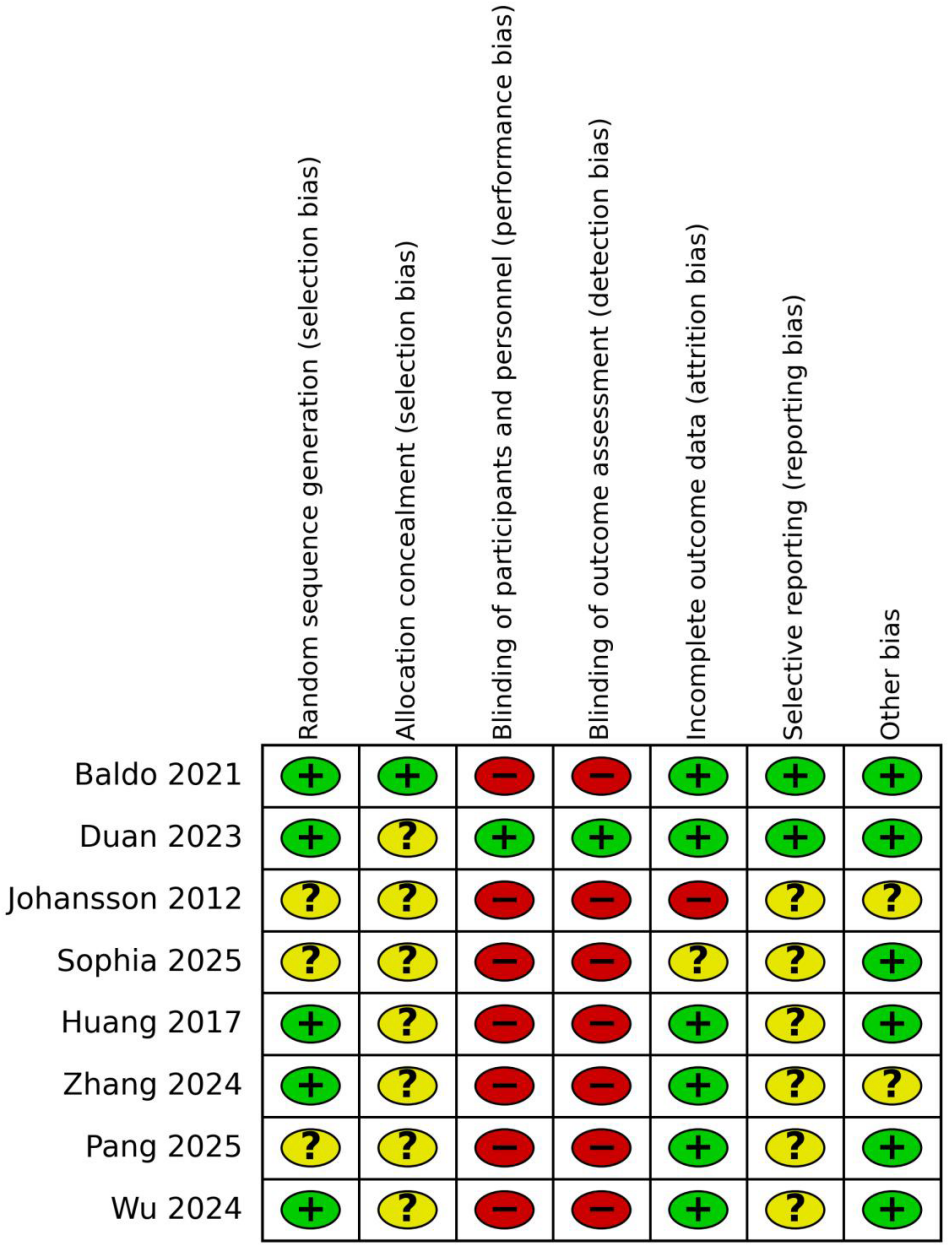
Risk of bias for included studies.

The risk-of-bias assessments for the included studies are presented in **Figures 2**, **3**. For random sequence generation, five studies were judged as having a low risk of bias (32, 33, 36, 37, 39), whereas the remaining three were rated as unclear because the randomization procedures were not sufficiently described (34, 35, 38). For allocation concealment, only one study clearly reported an adequate method and was judged as low risk (32), while the remaining studies were assessed as having an unclear risk due to insufficient reporting (32–39). Performance bias and detection bias were the main sources of bias across the included studies. Except for one double-blind, sham-controlled trial (33), most studies were judged as having a high risk in these two domains because blinding of participants and personnel was difficult to implement in MBSR-based interventions (32, 34–39). With regard to incomplete outcome data, most studies were considered to have a low risk of bias because outcome data were generally complete (32, 33, 36–39), whereas two studies were assessed more cautiously due to participant attrition (34, 35). For selective reporting, two studies were judged as having a low risk because prespecified outcomes or registration information were available (32, 33), while the remaining studies were rated as unclear (34–39). In the domain of other bias, six studies were assessed as having a low risk (32, 33, 35, 36, 38, 39), whereas two studies were rated as unclear (34, 37). Overall, the major sources of bias were related to blinding, whereas random sequence generation and incomplete outcome data were generally of lower concern.

### 3.4 Meta-analysis

#### 3.4.1 Effects on depression

**Figure 4:**
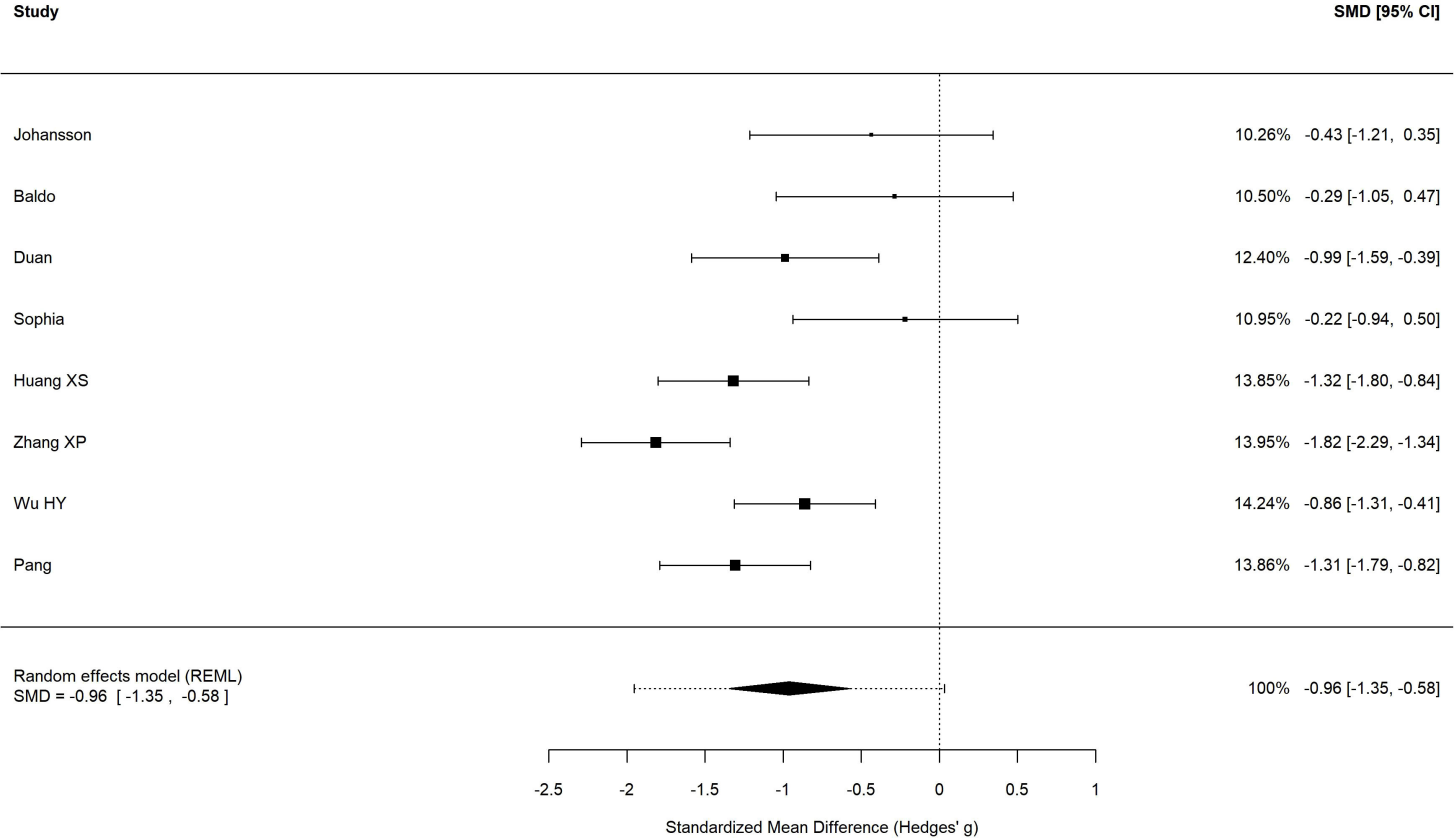
Forest plot of the effect of MBSR on depressive symptoms (random-effects model).

Eight studies (32–39) were included in the meta-analysis of depressive symptoms. All studies used standardized rating scales to assess depressive symptoms, and post-intervention data were included in the primary meta-analysis. A total of 469 participants were included, with 233 in the intervention group and 236 in the control group. Using a prespecified random-effects model, the pooled analysis showed that depressive symptom scores in the intervention group were significantly lower than those in the control group (standardized mean difference [SMD] = − 0.96, 95% confidence interval [CI]: − 1.35 to − 0.58, P < 0.001; **Figure 4**). Substantial heterogeneity was observed across studies (I² = 71.0%, 95% CI: 40.1%–86.0%; Q = 24.15, df = 7, P = 0.0011). The 95% prediction interval ranged from − 1.95 to 0.03, indicating that while the mean effect is beneficial, the true effect of MBSR on depressive symptoms may be negligible in some future settings.

Given the considerable between-study heterogeneity, subgroup analyses were conducted to explore potential sources.

#### 3.4.2 Subgroup analysis by geographic region

**Figure 5:**
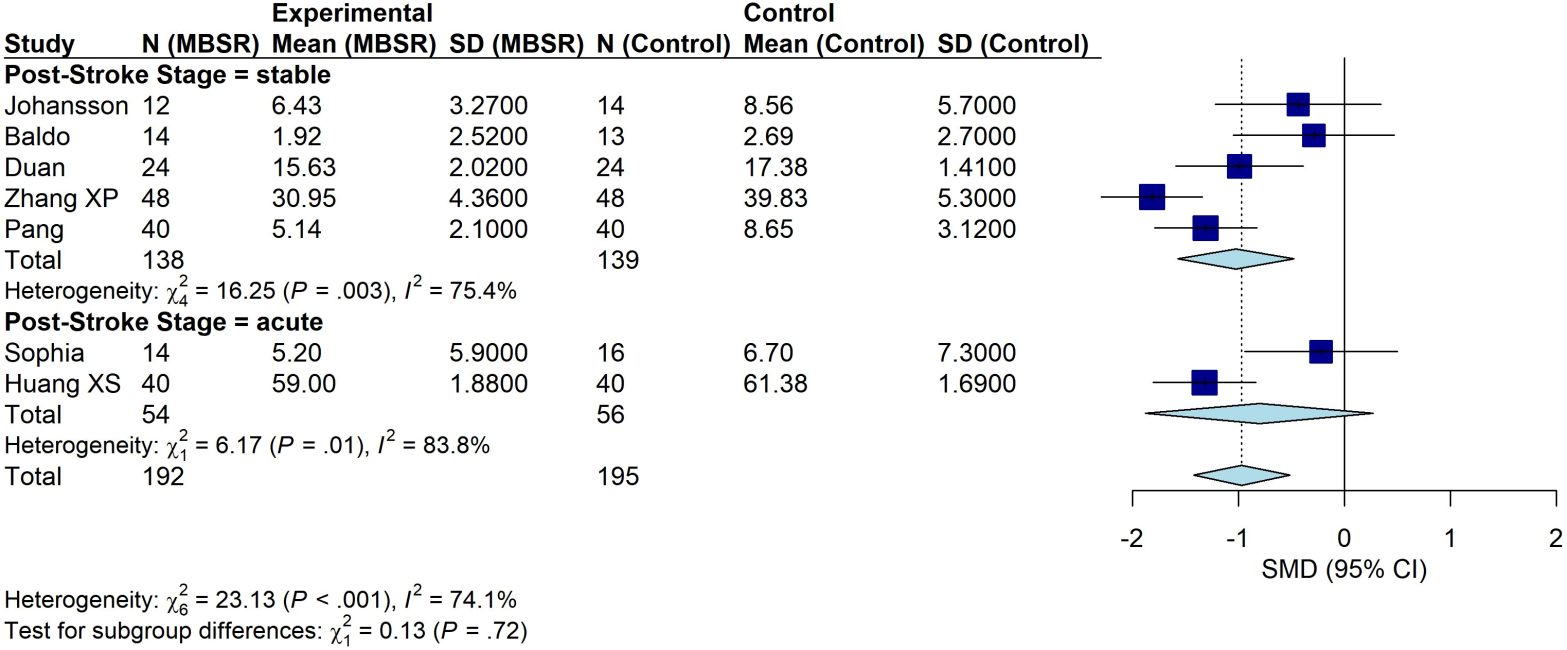
Subgroup analysis by geographic region.

Subgroup analyses were performed according to study location, stratifying trials into Asian and non-Asian groups. Five studies conducted in Asia (33, 36–39) included a total of 386 participants (193 in the intervention group and 193 in the control group). MBSR significantly reduced depressive symptoms compared with control conditions (SMD = − 1.27, 95% CI: − 1.60 to − 0.93, P < 0.001; I² = 55.7%; **Figure 5**).

Three studies conducted in non-Asian countries (32, 34, 35) included 83 participants (40 in the intervention group and 43 in the control group). No significant improvement was observed (SMD = − 0.31, 95% CI: − 0.74 to 0.13, P = 0.17; I² = 0%). The difference between subgroups was statistically significant (Q-between = 11.77, df = 1, P = 0.0006), suggesting that geographic region may moderate the effect of MBSR on depressive symptoms.

#### 3.4.3 Subgroup analysis by recruitment setting

**Figure 6:**
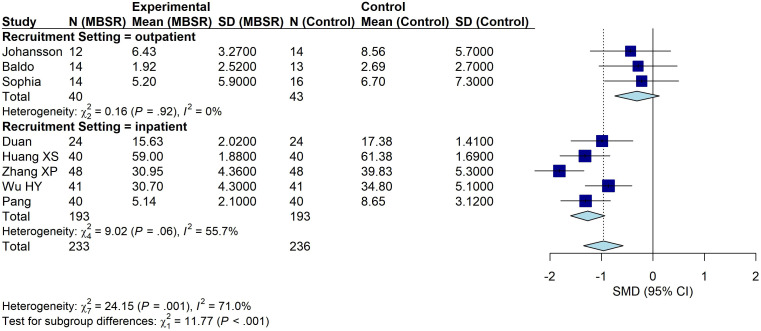
Subgroup analysis by recruitment setting.

Subgroup analyses were performed according to hospitalization status. Five studies delivered MBSR interventions to hospitalized stroke patients (33, 36–39), including 386 participants (193 in the intervention group and 193 in the control group). MBSR significantly reduced depressive symptoms (SMD = − 1.27, 95% CI: − 1.60 to − 0.93, P < 0.001; I² = 55.7%; **Figure 6**). Three studies enrolled non-hospitalized stroke patients (32, 34, 35), comprising 83 participants (40 in the intervention group and 43 in the control group). No significant improvement was observed (SMD = − 0.31, 95% CI: − 0.74 to 0.13, P = 0.17; I² = 0%). The difference between subgroups was statistically significant (Q-between = 11.77, df = 1, P = 0.0006), suggesting that the effect of MBSR may differ between inpatient and outpatient settings.

#### 3.4.4 Subgroup analysis by post-stroke stage

**Figure 7:**
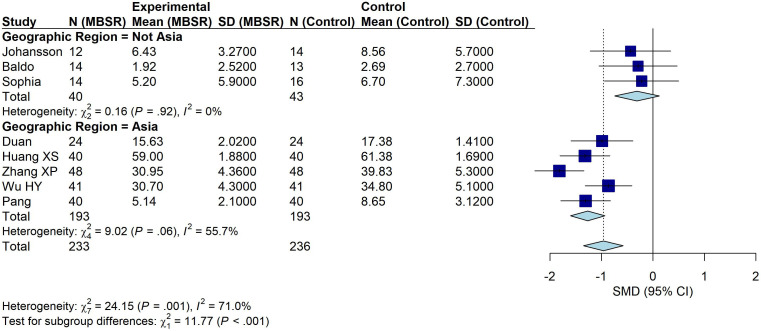
Subgroup analysis by post-stroke stage.

Subgroup analyses were conducted according to the post-stroke stage at which the intervention was delivered. Two studies conducted interventions during the acute phase (35, 36), including 110 participants (54 in the intervention group and 56 in the control group). The meta-analysis showed no significant difference between the MBSR and control groups (SMD = − 0.80, 95% CI: − 1.88 to 0.27, P = 0.14; I² = 83.8%; **Figure 7**). Five studies implemented interventions during the stable phase (32–34, 37, 38), including 281 participants (140 in the intervention group and 141 in the control group). Depressive symptoms were significantly improved in the intervention group compared with controls (SMD = − 1.02, 95% CI: − 1.57 to − 0.47, P < 0.001; I² = 75.4%). The difference between subgroups was not statistically significant (Q-between = 0.13, df = 1, P = 0.7204), indicating that the observed difference in effect sizes may be attributable to low statistical power in the acute-phase subgroup rather than a true moderating effect.

#### 3.4.5 Sensitivity analysis

A leave-one-out sensitivity analysis was conducted to assess the influence of individual studies on the pooled effect estimate. The SMD ranged from − 1.06 to − 0.85 after sequentially excluding each study, indicating that no single study disproportionately influenced the overall effect estimate. The direction and statistical significance of the pooled effect remained unchanged across all iterations (**Supplementary Figure S1**).

## 4 Discussion

The pathogenesis of PSD is thought to involve complex interactions between psychological and biological factors (40, 41). Neurological impairment after stroke and the long-term uncertainty associated with recovery may impose substantial psychological stress on patients. Concurrently, cerebral lesions can disrupt emotion-regulation networks and alter monoaminergic neurotransmission, including serotonergic and noradrenergic pathways (4). Against this background, non-pharmacological interventions—particularly psychotherapies—may offer advantages in reducing depressive symptoms and supporting rehabilitation, given their generally favorable safety profiles and potentially better acceptability and adherence (42). MBSR promotes non-judgmental awareness of present-moment experience through practices such as body scanning, mindfulness meditation, and gentle yoga, which may enhance acceptance of illness-related changes and facilitate psychological adjustment (43).

To evaluate efficacy, this study synthesized evidence from eight RCTs. Compared with control conditions, MBSR significantly reduced depressive symptoms in patients with PSD, suggesting a potential clinical benefit that warrants further investigation. Notably, all included trials were published after 2012, indicating that the available evidence is relatively recent. To our knowledge, this is the first meta-analysis to specifically assess MBSR as a standalone intervention for PSD, as prior meta-analyses have either combined MBSR with other mindfulness-based approaches or focused on broader populations. Nevertheless, substantial between-study heterogeneity was observed; therefore, the pooled estimates should be interpreted with caution. This heterogeneity may be attributable to several factors, including the frequent use of self-reported outcome measures (which are susceptible to reporting bias) and the limited feasibility of blinding in psychological interventions, which may introduce performance bias and potentially inflate effect estimates. Thus, although the pooled effect was statistically significant, its clinical relevance and generalizability should be confirmed in rigorously designed, high-quality trials. An SMD of −0.96 corresponds to a reduction of approximately 4–6 points on the Hamilton Depression Rating Scale (17-item version), which is considered a moderately clinically meaningful improvement.

Mechanistic research on the antidepressant effects of MBSR has largely examined changes in functional brain networks and structural neuroplasticity. Existing studies suggest that MBSR may modulate both neural circuit function and brain structure. At the network level, MBSR has been proposed to reduce hyperactivity within the default mode network, which has been implicated in mind-wandering, rumination, and affective disturbances such as depression and anxiety (44). In parallel, MBSR may enhance connectivity among regions of the executive control network, thereby supporting attentional control and emotion regulation (44). At the structural level, Lazar reported greater cortical thickness in regions involved in attention and sensory processing among individuals with long-term mindfulness training (45). These differences appeared more pronounced in older adults, suggesting that mindfulness-based training may help attenuate age-related cortical thinning (46).

Subgroup analyses indicated that effect estimates differed by region. In trials conducted in Asia, MBSR produced a larger reduction in depressive symptoms, whereas the effect was not statistically significant in trials conducted outside Asia (SMD = −0.31, 95% CI: −0.74 to 0.13). The difference between Asian and non-Asian subgroups was statistically significant (Q-between = 11.77, df = 1, P = 0.0006), suggesting that geographic region may moderate the effect of MBSR. Several factors may contribute to this pattern, including cultural context, intervention acceptability, and differences in healthcare delivery. MBSR is a standardized, structured, group-based psychotherapy derived from mindfulness practice (47). Mindfulness practices have historical roots in Buddhist traditions and emphasize nonjudgmental awareness of present-moment experience and openness to change (48, 49). In some Asian contexts, greater cultural familiarity with mindfulness-related concepts may facilitate engagement and increase perceived relevance. Supporting this possibility, a 2025 study of 445 Asian Americans and 265 European Americans reported higher acceptance of the “observing” and “nonjudging” facets among Asian participants during the intervention (50). Such cultural familiarity may have contributed to the larger effects observed in Asian studies, although this hypothesis requires confirmation in future research designed to directly assess cultural moderators. Nevertheless, evidence for cultural moderation remains limited; multicenter randomized controlled trials and implementation studies are warranted to clarify how cultural factors shape engagement and clinical outcomes.

In addition, our findings suggest that the effects of MBSR may differ by post-stroke stage. In subgroup analyses, MBSR initiated during the stable phase after stroke significantly reduced depressive symptoms (SMD = −1.02, 95% CI: −1.57 to −0.47), whereas the pooled effect was not statistically significant when the intervention was delivered during the acute phase (SMD = −0.80, 95% CI: −1.88 to 0.27). The difference between stable-phase and acute-phase subgroups was not statistically significant (Q-between = 0.13, df = 1, P = 0.7204), indicating that the observed difference in effect sizes may be attributable to low statistical power in the acute-phase subgroup (k = 2) rather than a true moderating effect. Stroke recovery is commonly described as progressing through acute, subacute, and stable phases, and depressive symptoms can occur across all stages (51). During the acute or subacute phases, clinical instability, evolving neurological deficits, and a limited capacity to participate in structured psychological programs may reduce adherence and diminish treatment effects (52–54). Accordingly, many trials preferentially implement psychotherapeutic interventions during more stable phases. Nevertheless, depression in the acute and subacute stages remains clinically important. Future studies should assess the feasibility and safety of delivering psychotherapeutic interventions earlier after stroke and identify the optimal timing and formats tailored to these stages.

Several limitations should be acknowledged. First, the small number of RCTs may limit the precision and robustness of the pooled estimates. Second, substantial heterogeneity indicates that clinical and methodological diversity—including differences in participant characteristics, intervention delivery, and comparator conditions—likely contributed to residual variability. Third, blinding was limited and is often difficult to implement in psychological interventions, which may have introduced performance bias. Fourth, the lack of long-term follow-up in most trials precluded a rigorous evaluation of the durability of MBSR effects. Fifth, although a leave−one−out sensitivity analysis suggested that no single study disproportionately influenced the pooled estimate (SMD range −1.06 to −0.85), the small number of studies precluded more robust sensitivity analyses, such as meta−regression. Sixth, grey literature was excluded to maintain methodological homogeneity, which may have introduced some risk of publication bias.

Despite these limitations, this meta-analysis provides preliminary evidence that MBSR can alleviate depressive symptoms in PSD and may serve as an adjunct to comprehensive post-stroke mental health care. Future research should prioritize large, multicenter, methodologically rigorous RCTs with standardized outcome assessment and extended follow-up to confirm effectiveness and durability and to identify subgroups most likely to benefit. Overall, the current evidence suggests that MBSR may be particularly suitable for patients in the stable post-stroke phase. From a clinical perspective, MBSR may be considered a feasible non−pharmacological adjunct for patients in the stable phase after stroke, particularly in settings where cultural familiarity with mindfulness practices may enhance engagement. However, given the low certainty of evidence, treatment decisions should be individualized, and patients should be informed that the magnitude of benefit remains uncertain.

## Data Availability

The original contributions presented in the study are included in the article/**Supplementary Material**. Further inquiries can be directed to the corresponding author.
