# Supplementary figures and images for: Effectiveness of mindfulness-based stress reduction for depression in post-stroke patients: a systematic review and meta-analysis
Source: Front Psychiatry. 2026 May 8;17:1809626. doi: 10.3389/fpsyt.2026.1809626 (PMC13193968; doi:10.3389/fpsyt.2026.1809626)

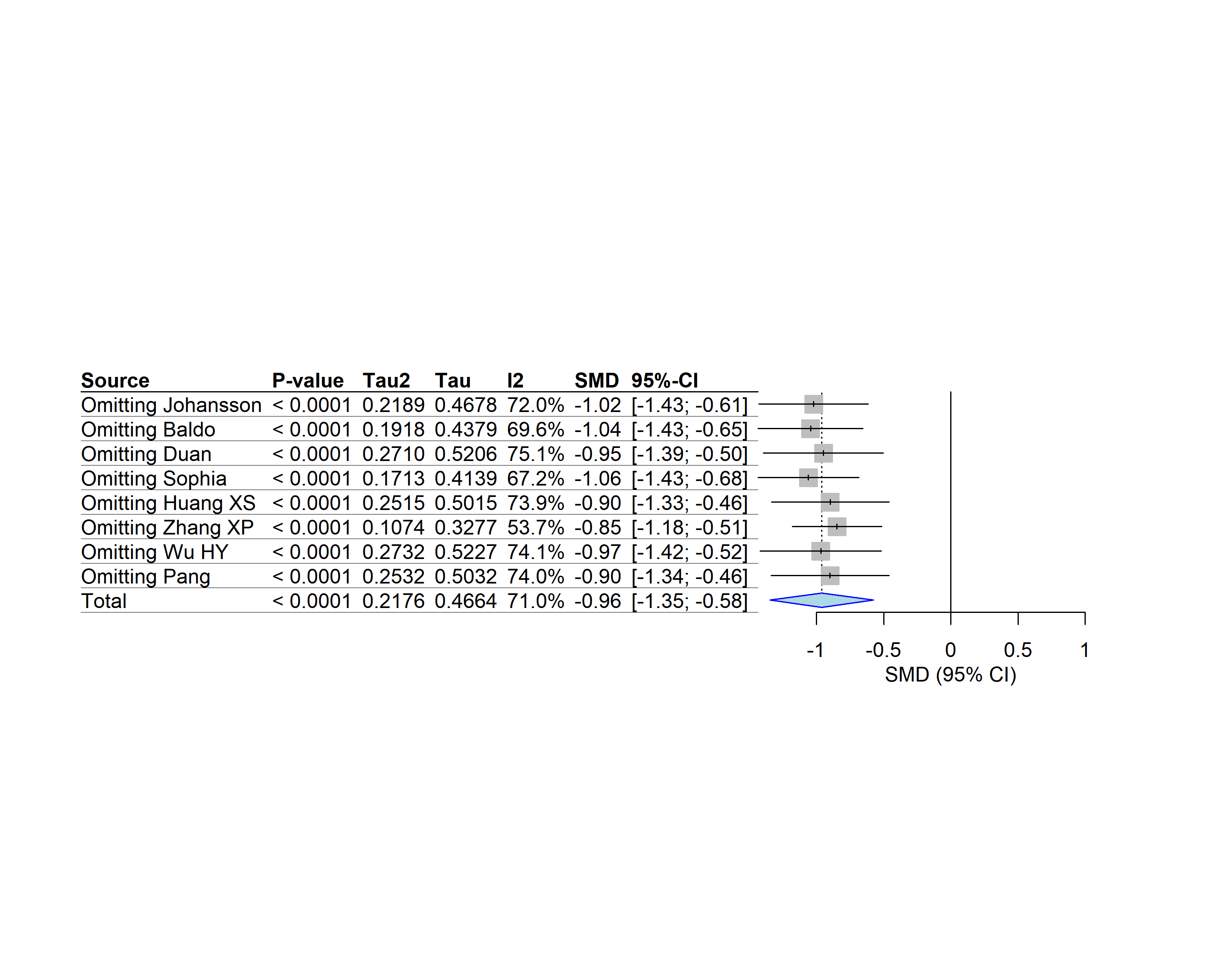

Supplement: Supplementary file 1 [file Image1.jpeg]
