# Supplementary material for: Effectiveness of mindfulness-based stress reduction for depression in post-stroke patients: a systematic review and meta-analysis
Source: Front Psychiatry. 2026 May 8;17:1809626. doi: 10.3389/fpsyt.2026.1809626 (PMC13193968; doi:10.3389/fpsyt.2026.1809626)
Supplement: Supplementary file 2 [file Table1.docx]

**Supplementary Table S1. Studies excluded due to full-text unavailability**

| Study identifier | Title | Reason for unavailability |
| --- | --- | --- |
| NCT02687048 | Mindful Meditation for Chronic Stroke | Full text unavailable; study registered on [ClinicalTrials.gov](https://clinicaltrials.gov/) but no published manuscript identified |
| NCT02792608 | Mindfulness-Based Therapy for Brain Tumour Survivors | Full text unavailable; study registered on [ClinicalTrials.gov](https://clinicaltrials.gov/) but no published manuscript identified |
| NCT03659409 | Stroke of Mindfulness: Investigating Physiological and Psychological Well-being (SOM) | Full text unavailable; study registered on [ClinicalTrials.gov](https://clinicaltrials.gov/) but no published manuscript identified |
| NCT04302493 | Mindfulness Based Stress Reduction and Post-Stroke Cognition | Full text unavailable; study registered on [ClinicalTrials.gov](https://clinicaltrials.gov/) but no published manuscript identified |
| NCT03910855 | Impact of Mindfulness on Psychological Well-being of Stroke Survivors and Their Caregivers | Full text unavailable; study registered on [ClinicalTrials.gov](https://clinicaltrials.gov/) but no published manuscript identified |
| Not identified | Clinical Effectiveness of using Mindfulness-Based Cognitive Therapy to Improve Coping and Quality of Life for Stroke Survivors | Full text unavailable after contacting authors |

### **Supplementary Table S2. Intervention characteristics of included studies**

| Study (year) | MBSR format | Duration (weeks) | Fidelity assessment | Control type |
| --- | --- | --- | --- | --- |
| Johansson, 2012 | Standard | 8 | Not reported | No treatment |
| Baldo, 2021 | Standard | 8 | Expert rating (4.5–5/5) | Brain Health Education |
| Duan, 2023 | Modified (6 sessions, no retreat) | 6 | Not reported | Sham rTMS stimulation |
| Sophia, 2025 | Modified (online, shorter sessions) | 8 | Not reported | Stroke Support Group |
| Huang, 2017 | Standard | 8 | Not reported | Usual care |
| Zhang, 2024 | Not specified | Not reported | Not reported | Usual care |
| Wu, 2024 | Not specified | 6 | Not reported | Usual care |
| Pang, 2025 | Standard | 8 | Not reported | Usual care |

### **Supplementary Table S3. Extracted data for depression outcome**

| Author (year) | Intervention group | Control group | Scale | | |
| --- | --- | --- | --- | --- | --- |
|  | N | Mean (SD) | N | Mean (SD) |  |
| Johansson, 2012 | 12 | 6.43 (3.27) | 14 | 8.56 (5.70) | CPRS |
| Baldo, 2021 | 14 | 1.92 (2.52) | 13 | 2.69 (2.70) | GDS |
| Duan, 2023 | 24 | 15.63 (2.02) | 24 | 17.38 (1.41) | HAMD‑17 |
| Sophia, 2025 | 14 | 5.20 (5.90) | 16 | 6.70 (7.30) | PHQ |
| Huang, 2017 | 40 | 59.00 (1.88) | 40 | 61.38 (1.69) | SDS |
| Zhang, 2024 | 48 | 30.95 (4.36) | 48 | 39.83 (5.30) | SDS |
| Wu, 2024 | 41 | 30.70 (4.30) | 41 | 34.80 (5.10) | SCL‑90 |
| Pang, 2025 | 40 | 5.14 (2.10) | 40 | 8.65 (3.12) | HAMD‑17 |

****Abbreviations****: CPRS, Comprehensive Psychopathological Rating Scale; GDS, Geriatric Depression Scale; HAMD‑17, Hamilton Depression Rating Scale (17‑item); PHQ, Patient Health Questionnaire; SDS, Self‑rating Depression Scale; SCL‑90, Symptom Checklist‑90.
